# Field-Induced Slow Magnetic Relaxation in Pentacoordinate Co(II) Complexes: Tuning Magnetic Anisotropy Through Halide Substitution

**DOI:** 10.3390/molecules30112295

**Published:** 2025-05-23

**Authors:** Hong-Yao Guo, Wei-Quan Lin, Ji-Dong Leng

**Affiliations:** School of Chemistry and Chemical Engineering/Institute of Clean Energy and Materials, Guangzhou Higher Education Mega Center, Guangzhou University, No. 230 Wai Huan Xi Road, Guangzhou 510006, China; palestarlight@163.com

**Keywords:** single-ion magnets, cobalt(II) complexes, pentacoordinate geometry, magnetic anisotropy, halide substitution, bidentate phosphine ligands

## Abstract

We report the synthesis, structural characterization, and magnetic properties of three pentacoordinate Co(II) complexes [CoX(dppb)_2_]X (X = Cl (**1Cl**), Br (**2Br**), and I (**3I**)) supported by the bidentate phosphine ligand 1,2-bis(diphenylphosphino)benzene (dppb). Single-crystal X-ray diffraction reveals that all three complexes adopt similar vacant octahedron (*C*_4v_) geometries with the halide ligand in one axial position. Magnetic studies demonstrate that these complexes exhibit field-induced slow magnetic relaxation behaviors, with positive *D* values of 25.3(2), 21.6(1), and 19.4(2) cm^−1^ for **1Cl**, **2Br**, and **3I**, respectively. Detailed analysis of the relaxation dynamics reveals that Raman processes dominate at higher temperatures, with systematic variations in relaxation parameters across the series. The systematic variations in magnetic anisotropy and slow magnetic relaxation behaviors of the three complexes correlate with the decreasing electronegativity of the halide ligands.

## 1. Introduction

Single-molecule magnets (SMMs) have garnered significant attention in recent decades due to their potential applications in high-density magnetic memory, quantum computing, and molecular spintronics [1,2]. These molecular materials exhibit the remarkable ability to retain their magnetization below certain temperatures without requiring long-range magnetic ordering [3]. The performance of SMMs is primarily governed by the spin-reversal energy barrier (*U*_eff_), which determines the difficulty of magnetization reversal and is directly related to the magnetic anisotropy and ground state spin. The reversal of magnetization is driven by spin–lattice relaxation and quantum tunneling magnetization (QTM). To achieve practical applications, significant efforts have been directed toward designing SMMs with higher energy barriers and blocking temperatures (*T*_B_) [4].

Among the various types of SMMs, single-ion magnets (SIMs), which contain only one paramagnetic metal center, have emerged as a rapidly developing subclass [5]. The magnetic properties of these complexes arise from a single ion in an appropriate ligand field, making them particularly attractive for fundamental studies of magnetic anisotropy. While lanthanide-based SIMs have demonstrated remarkable magnetic properties due to their strong spin–orbit coupling and significant magnetic anisotropy, transition metal-based SIMs have also attracted considerable interest as their magnetic properties can be systematically tuned through ligand field engineering [6].

Among various transition metal ions, cobalt(II) is considered an exceptional candidate for the design and synthesis of SIMs due to its distinctive electronic properties [7,8]. The high-spin Co(II) ion (3d^7^, *S* = 3/2) possesses significant unquenched orbital angular momentum in certain coordination environments [9], which contributes to large magnetic anisotropy—a crucial prerequisite for effective SIMs [5]. Moreover, Co(II) ions exhibit a half-integer spin ground state that theoretically suppresses QTM at zero field according to Kramers’ theorem, although weak QTM effects often remain due to hyperfine interactions and dipolar coupling [10,11]. Notably, Co(II) has 100% natural abundance of nuclear spin *I* = 7/2, which introduces significant hyperfine coupling that could create a local transverse magnetic field at the Co(II) center [12]. This hyperfine coupling splits the electronic energy levels into multiple avoided level crossings, creating tunneling pathways that facilitate QTM, thereby resulting in rapid magnetic relaxation.

The magnetic behavior of Co(II)-based SIMs can be systematically tuned through coordination geometry manipulation, with various coordination numbers from two to eight having been explored. Two-coordinate linear Co(II) complexes typically exhibit exceptional magnetic anisotropy and impressive energy barriers due to minimal ligand field effects, though their extreme air sensitivity limits practical applications [8,13]. Four-coordinate Co(II) complexes, particularly those with tetrahedral geometry, represent an important class of SIMs that balance significant magnetic anisotropy with improved stability [14,15,16,17,18]. Six-coordinate octahedral Co(II) complexes have also been widely studied, though they typically exhibit weaker magnetic anisotropy due to more extensive ligand field splitting [10,19,20,21]. In contrast, pentacoordinate Co(II) complexes remain relatively underexplored in the field of SIMs, despite their potential for unique magnetic behavior [22,23,24,25,26]. The synthetic challenges associated with stabilizing five-coordinate geometries, whether square pyramidal or trigonal bipyramidal, have limited their development. However, these intermediate coordination environments may offer an optimal balance between orbital angular momentum preservation and structural stability, potentially leading to enhanced magnetic properties through fine-tuning of the axial and equatorial ligand field components. On the other hand, the sensitivity of Co(II) to subtle changes in its coordination environment also provides an excellent platform for establishing magneto-structural correlations [5].

Bidentate phosphine and phosphine oxide ligands have proven to be versatile tools for designing transition metal SIMs with controlled coordination environments [15,27]. These ligands offer several advantages for the rational synthesis of SIMs with specific coordination numbers and symmetries [28]. The structural versatility of these ligands allows for fine-tuning of their bite angle, flexibility, and steric hindrance, which directly impacts the coordination geometry around the metal center [29]. For instance, our group has employed rigid bidentate phosphine oxide ligands to create lanthanide complexes with *D*_5h_ symmetry and equatorial compressed ligand fields, demonstrating how ligand choice can dictate symmetry [30]. By adjusting the groups connecting the phosphine or phosphine oxide moieties, researchers can systematically modify the coordination sphere from tetrahedral to octahedral geometries. Additionally, the steric bulk of substituents on the phosphorus atoms can effectively limit coordination numbers, enabling the isolation of low-coordinate species that often exhibit enhanced magnetic anisotropy [31]. The strategic design of bidentate phosphine ligands thus offers a promising pathway for developing transition metal SIMs with tailored magnetic properties through precise control of coordination geometry and symmetry.

In this work, we present a systematic investigation of a series of pentacoordinate Co(II) complexes [CoX(dppb)_2_]X (X = Cl (**1**), Br (**2**) and I (**3**)) supported by the bidentate phosphine ligand 1,2-bis(diphenylphosphino)benzene (dppb). Through careful synthetic design, we have successfully isolated three structurally similar complexes that differ only in their halide anions (Cl, Br, and I). The molecular structures of these complexes were determined by single-crystal X-ray diffraction, revealing the pentacoordinate geometry around the Co(II) center that has been stabilized by the bidentate phosphine ligand. Direct current (DC) and alternating current (AC) magnetic susceptibility measurements were conducted and demonstrated field-induced slow magnetic relaxation behavior characteristic of SMMs. The systematic variation in halide ligands allowed us to examine the subtle effects of axial ligand substitution on magnetic anisotropy and relaxation dynamics.

## 2. Experimental

### 2.1. General Remarks

All chemicals were commercially available and used as received without further purification. The C, H, and N microanalyses were carried out with an Elementar Vario EL Cube elemental analyzer (Elementar, Langenselbold, Germany). The FT-IR spectra were recorded from KBr pellets in the range 4000–400 cm^–1^ on a Bruker TENSOR II spectrometer (Billerica, MA, USA). The powder XRD patterns were recorded on a Bruker D8 X-ray diffractometer (Billerica, MA, USA, CuK*α*). Thermogravimetric analyses were carried out on a NETZSCH TG209F3 thermogravimetric analyzer (NETZSCH, Selb, Germany) in the range of 30–800 °C, with a heating rate of 10 K min^−1^ under N_2_ atmosphere.

Magnetic measurements: Magnetic measurements were performed using a Quantum Design MPMS3 SQUID magnetometer (Quantum Design, San Diego, CA, USA). Polycrystalline samples with a mass of ca. 25 mg were embedded in Vaseline to prevent torquing and were then packed in plastic film. The samples were then loaded into the magnetometer with plastic straws. DC magnetic susceptibility measurements were performed in the temperature range of 2–300 K under an applied field of 1000 Oe. The field-dependent magnetization measurements were performed at 2.0, 3.0, and 5.0 K, under the field range of 0–7 Tesla. AC magnetic susceptibility data measurements were performed at frequencies between 1 and 999 Hz. Data were corrected for the non-magnetic contribution from the sample and the sample holder.

#### X-Ray Structure Determination

The intensity data were recorded on a Rigaku XtaLAB Synergy system with Cu *K*_α_ (*λ* = 1.54178 Å, for **2**) and Mo *K*_α_ radiation (*λ* = 0.71073 Å, for **1** and **3**). Absorption correction based on symmetry equivalent reflections was applied using the SADABS program. The SQUEEZE [32] subroutine of the PLATON V1.19 [33] software was used to remove the scatering from the highly disordered solvent molecules. The calculations suggest 96, 100, and 106 electrons per unit cell, for **1**, **2,** and **3**, respectively. The values are consistent with the presence of two methanol (CH_3_OH) and two H_2_O per asymmetric unit, which account for 112 electrons per unit cell. Thermogravimetric analysis (Figure S3) of these complexes confirms the presence of lattice solvent molecules, providing additional support for the structural assignments. The resulting new HKL files were used to further refine the structures. Structures were solved with the direct method and refined with full-matrix least-squares (SHELX-2014/7) [34]. All non-hydrogen atoms were refined anisotropically by full-matrix least-squares on *F*^2^ using the SHELXTL program. Anisotropic thermal parameters were assigned to all non-hydrogen atoms. Hydrogen atoms of organic ligands were generated by the riding mode. Structure plots and space-filling diagrams were produced with DIAMOND 3.1 and POV-Ray v3.7 [35]. X-ray crystallographic files for the structure have been deposited in the Cambridge Crystallographic Data Centre (CCDC) with no. of 2446387-2446389. These data can be obtained free of charge via https://www.ccdc.cam.ac.uk/structures/ (accessed on 21 May 2025) (or from the Cambridge Crystallographic Data Centre, 12 Union Road, Cambridge CB21EZ, UK; fax (+44)1223-336-033 or e-mail deposit@ccdc.cam.ac.uk).

### 2.2. Synthesis

**[CoCl(dppb)_2_]Cl·2MeOH·2H_2_O (1)**: A mixture of dppb (112 mg, 0.25 mmol), CoCl_2_·6H_2_O (48 mg, 0.2 mmol) and 8 mL MeOH was sealed in a 25 mL Teflon-lined, stainless-steel vessel and heated at 120 °C for 72 h, and then cooled slowly to room temperature at 5 °C /h. Purple crystals were obtained (yield ca. 65% based on Co). Calculated elemental analysis values for C_62_H_60_Cl_2_CoO_4_P_4_ (%): C 66.31, H 5.39. Found values: C 66.28, H 5.32. IR (KBr, cm^−1^): *v* = 3050(s), 1625(m), 1480(s), 1433(s), 1309(s), 1250(s), 1184(s), 1187(s),1162(s), 1090(s), 1046(s),997(s), 741(s), 692(s), 524(s).

**[CoBr(dppb)_2_]Br·2MeOH·2H_2_O (2)**: The procedure was the same as that employed for **1**, except that CoBr_2_·H_2_O (47 mg, 0.2 mmol) was employed as cobalt salt. Purple crystals were obtained (yield ca. 62% based on Co). Calculated elemental analysis values for C_62_H_60_Br_2_CoO_4_P_4_ (%): C 61.45, H 4.99. Found values: C 61.37, H 4.93. IR (KBr, cm^−1^): *v* = 3051(s), 1620(m), 1482(s), 1436(s), 1312(s), 1252(s), 1185(s), 1190(s),1159(s), 1090(s), 1045(s),997(s), 733(s), 692(s), 520(s).

**[CoI(dppb)_2_]I·2MeOH·2H_2_O (3)**: The procedure was the same as that employed for **1**, except that CoI_2_ (62 mg, 0.2 mmol) was employed as a cobalt salt. Purple crystals were obtained (yield ca. 45% based on Co). Calculated elemental analysis values for C_62_H_60_CoI_2_O_4_P_4_ (%): C 57.02, H 4.63. Found values: C 56.93, H 4.56. IR (KBr, cm^−1^): *v* = 3048(s), 1621(m), 1485(s), 1431(s), 1316(s), 1244(s), 1184(s), 1188(s),1162(s), 1088(s), 1045(s),998(s), 741(s), 690(s), 525(s).

## 3. Results and Discussion

### 3.1. Crystal Structures

**Figure 1:**
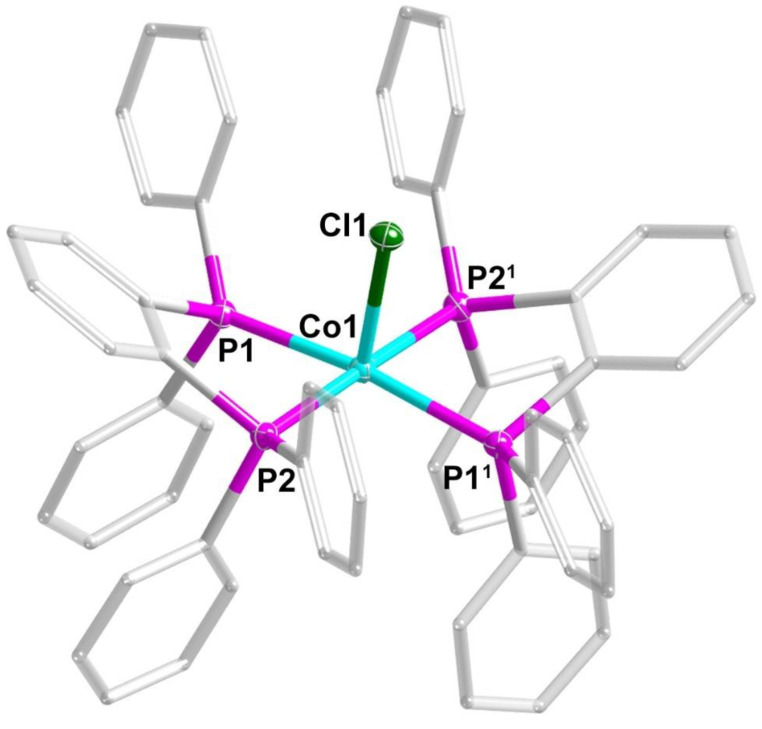
Structure of the [CoCl(dppb)_2_]^+^ coordination cation of **1Cl**. Hydrogen atoms are omitted for clarity. Displacement ellipsoids of selected atoms are set at the 50% probability level. Color code: Co, turquoise; C, gray; P, pink; Cl, green. Symmetric code: ^1^ 1/2 − *X*, +*Y*, 1/2 − *Z*.

The molecular structures of the three pentacoordinate Co(II) complexes were determined by single-crystal X-ray diffraction analysis. The three complexes are isostructural, differing only in the coordinated halide anion (Cl, Br, and I). In the cationic Co(II) complex, the Co center is coordinated with one halogen anion and two chelating bidentate phosphine ligands, dppb, resulting in a coordination number of five (Figure 1).

The Co-X bond lengths are 2.3937(11), 2.5295(7), and 2.7014(9) Å for **1Cl**, **2Br,** and **3I**, respectively, reflecting the increasing ionic radii of the halide anions. The two bidentate dppb ligands coordinate to the Co(II) center through their phosphorus atoms, with Co-P bond distances ranging from 2.2516(8) to 2.2931(8) Å. These values are consistent with typical Co(II)-halogen ions and Co(II)-P bond lengths observed in related cobalt complexes [36,37]. The bite angles of the chelating dppb ligands (P1-Co-P2) are 81.98(3)° (**1Cl**), 82.02(3)° (**2Br**), and 82.15(4)° (**3I**), which contribute to the geometric constraints around the metal center and help stabilize the pentacoordinate environment. The dihedral angle between the two chelating P-Co-P planes for the three complexes is 9.05(5), 8.85(5), and 9.46(7)°, respectively, indicating a slight twisting of the two chelate rings relative to each other.

To quantitatively assess the coordination geometry around the Co(II) centers, continuous shape measures (CShM) [38] calculations were employed (Table 1) and suggested that the local geometries of the Co(II) ions are close to ideal vacant octahedron (*C*_4v_), with obtained values of 0.63020, 0.80706, and 1.16116 for **1Cl**, **2Br,** and **3I** (Table 1), respectively. In this arrangement, the four phosphorus atoms from the two bidentate dppb ligands occupy the equatorial positions, while the halide anion occupies one of the axial sites. The Co(II) ion is positioned very close to the P4 basal plane, resulting in a pentacoordinate geometry that can be visualized as an octahedron with one vacant coordination site. Notably, the CShM values for spherical square pyramidal geometry (*C*_4v_) are also quite small for all three complexes. In fact, for complex **3I**, the CShM value for the spherical square pyramid (1.12414) is slightly smaller than that for the vacant octahedron (1.16116). Despite this numerical difference, we have classified the local geometry of **3I** as a vacant octahedron to better reflect the structural similarities among the three complexes. This classification is supported by an examination of the bond angles in the coordination sphere. The adjacent P-Co-P bond angles in all three complexes are remarkably similar and closer to 90°, which aligns better with the ideal vacant octahedral geometry (which has 90° angles) than with the ideal spherical square pyramidal geometry (which has angles of approximately 105°). The progressive increase in CShM values across the series from **1Cl** to **3I** can be attributed primarily to the elongation of the Co-halide bond length rather than to any significant changes in the overall coordination geometry.

Despite these differences in the Co-X bond lengths, the overall coordination geometry remains remarkably similar across the series, with only minor variations in bond angles and distances. As illustrated in Figure S1, when the structures of the three complex cations are superimposed with the Co(II) ions and Co-X bonds aligned, the four phosphorus atoms from the dppb ligands exhibit nearly perfect overlap. This structural invariance, apart from the Co-X bond length, provides an excellent platform for investigating the specific influence of the halide ligand on the magnetic properties of these pentacoordinate Co(II) complexes while minimizing other structural variables.

Intramolecularly, two pairs of π–π stacking interactions are observed between the phenyl rings of the dppb ligands (Figure S2 and Table S3). One pair exhibits relatively strong interactions, while the other pair shows considerably weaker π–π stacking. The stronger π–π stacking interaction occurs between phenyl rings from two different dppb ligands (the yellow ones in Figure S2). This interaction likely contributes to the overall stability of the molecular structure and helps maintain the rigid coordination environment around the Co(II) center. In contrast, the second type of π-π stacking is considerably weaker and exists between phenyl rings of the same dppb ligand (the turquoise ones in Figure S2). This interaction is particularly weak in complex **3I**, where the centroid-to-centroid distance measures 3.946 Å. At this distance, the interaction is extremely weak or could even be considered negligible as a true π–π stacking interaction [39]. For comparison, in related complexes, significant π–π stacking interactions typically exhibit centroid-to-centroid distances in the range of 3.716–3.850 Å [36,37,40]. Interestingly, the parameters of both stacking interactions are notably affected by the halide substitution, suggesting that the electronic influence of the halide extends beyond the immediate coordination sphere to affect the arrangement of the peripheral aromatic rings.

It is noteworthy that the structures of our three complex cations bear similarities to the previously reported [CoCl(dppb)_2_]·ClO₄ (**4**) [26]. A detailed comparison between the Cl-coordinated complexes **1Cl** and **4** reveals that while most bond lengths and angles are very similar, there are subtle structural distinctions (Table 2). The most notable difference is observed in the P-Co-P bond angle at the diagonal positions of the P₄ basal plane. For complex **1Cl**, this angle measures 170.99(5)°, whereas for complex **4**, it is 169.02(3)°—a difference of nearly 2°. This angular variation consequently leads to a larger dihedral angle between the two chelating P-Co-P planes in complex **4**. In our complexes **1Cl**–**3I**, the Co(II) ion is positioned above the P₄ basal plane at distances ranging from 0.1063(11) to 0.1108(7) Å. In contrast, the Co(II) ion in complex **4** is located below the P₄ basal plane. Furthermore, complex **4** exhibits only one type of intramolecular π–π interaction, whereas our complexes display two distinct types of π–π stacking interactions (one strong and one weak) as previously discussed. These structural differences collectively indicate that complex **4** possesses a more distorted coordination environment compared to our series of complexes. Similar to observations in other cobalt complexes, where subtle distortions from ideal geometry can significantly impact magnetic behavior, these minor structural variations between complex **4** and our series **1Cl**–**3I** likely account for their different slow magnetic relaxation behaviors [21,41,42].

In the crystal lattice, each cationic Co(II) complex is accompanied by a halide counterion (Cl^−^, Br^−^, or I^−^) to balance the charge. Despite the presence of these anions, no significant intermolecular supramolecular interactions are observed between the complex cations or between the cations and the counter anions. In contrast, the perchlorate anion in complex **4**, with its four oxygen atoms, provides multiple hydrogen bond acceptor sites that can interact with C-H groups from the phenyl rings of the dppb ligands. These extensive C-H···O hydrogen bonding networks could pull the phenyl rings into specific orientations, consequently affecting the P-Co-P bond angles and the overall coordination geometry.

The crystal packing reveals that the cationic Co(II) complexes are well-separated from each other, with the shortest Co···Co distances being 9.4572(5), 9.489(1), and 9.590(1) Å for **1Cl**, **2Br,** and **3I**, respectively. This significant metal–metal separation suggests that intermolecular magnetic interactions should be negligible, allowing for the observation of single-ion magnetic properties [26].

### 3.2. DC Magnetic Measurements

**Figure 2:**
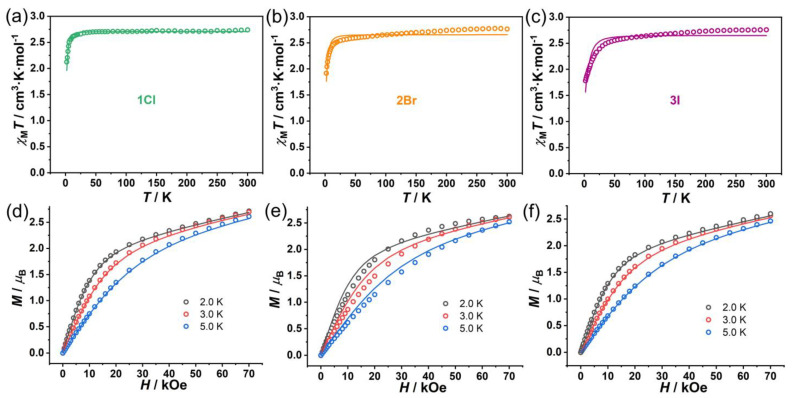
Temperature-dependent *χ*_M_*T* data (**a**–**c**) and field-dependent magnetization data (**d**–**f**) of **1Cl** (left), **2Br** (middle), and **3I** (right). Open circles and solid lines represent the experimental data and the fitting values using PHI, respectively.

The phase purity of the bulk samples was confirmed through powder X-ray diffraction measurements, which demonstrated excellent agreement between the experimental patterns and those simulated from the single-crystal structure data (Figure S4). DC magnetic susceptibility measurements were conducted on polycrystalline samples of complexes **1Cl**–**3I** over the temperature range of 2–300 K under an applied field of 0.1 T (Figure 2a–c). The room temperature *χ*_M_*T* values for **1Cl**, **2Br**, and **3I** are 2.72, 2.76, and 2.74 cm^3^ K mol^−1^, respectively. These values significantly exceed the spin-only value of 1.88 cm^3^ K mol^−1^ expected for a high-spin Co(II) center with *S* = 3/2 and *g* = 2, indicating substantial spin–orbit coupling effects characteristic of Co(II) ions [7]. As the temperature decreases, the *χ*_M_*T* products decline gradually until approximately 35 K, below which they decrease more rapidly, ultimately reaching values of 2.09, 1.90, and 1.78 cm^3^ K mol^−1^ for **1Cl**, **2Br**, and **3I**, respectively, at 2 K. This thermal behavior is typical for mononuclear Co(II) complexes with significant magnetic anisotropy arising from zero-field splitting (ZFS) of the *S* = 3/2 state, where positive *D* values lead to an *M*_S_ = ±1/2 Kramers doublet ground state [8]. Field-dependent magnetization measurements (*M* vs. *H*, Figure 2d–f) reveal that the magnetization increases sharply up to approximately 2 T, followed by a more gradual increase without reaching saturation even at the maximum applied field of 7 T. The maximum magnetization values at 2 K and 7 T are 2.71, 2.62, and 2.59 *μ*_B_ for **1Cl**, **2Br**, and **3I**, respectively.

While the overall static magnetic behaviors of the three complexes are qualitatively similar, indicating their common pentacoordinate Co(II) nature, the subtle differences observed in both the temperature-dependent *χ*_M_*T* curves and field-dependent magnetization data suggest variations in their magnetic anisotropy parameters. These differences likely arise from the electronic influence of the different halide ligands, as the coordination geometries remain nearly identical across the series. The progressive decrease in both the low-temperature *χ*_M_*T* values and the maximum magnetization values from **1Cl** to **3I** correlates with the increasing size and decreasing electronegativity of the halide ligands, suggesting a systematic trend in the magnetic anisotropy parameters.

The PHI [43] program was used to obtain ZFS parameters by concurrent fitting of *χ*_M_*T* vs. *T* and *M* vs. *H* plots, employing an isotropic *g* tensor according to the spin Hamiltonian: *H* = *μ*_B_*g*·*B*·*S* + *D*[*S*_z_^2^ − *S*(*S* + 1)/3] + *E*(*S*_x_^2^ − *S*_y_^2^) [15]. The *D* and *E* terms represent the axial and rhombic ZFS parameters, respectively. The best fitting results are summarized in Table 3. To verify the correct sign of the ZFS parameter, we also performed fittings of the DC magnetic plots with negative initial *D* values. However, these attempts yielded significantly poorer fits to the experimental data. Moreover, the results obtained with negative *D* values are physically unreasonable as they violate the general relation between *D* and *E* parameters, which requires |*D*/*E*| ≥ 3. Therefore, our fitting analysis strongly suggests that all three complexes possess transverse (positive *D*) magnetic anisotropies. This finding is consistent with the previously reported results for complex **4**, which exhibited a *D* value of 48.5(3) cm^−1^ [26]. Notably, complexes **1Cl**–**3I** display somewhat smaller *D* values compared to complex **4**, which can be attributed to the subtle structural differences discussed earlier. The variation in *D* values across our series likely reflects the electronic influence of the different halide ligands, as the coordination geometries remain remarkably similar.

As the electronegativity decreases from Cl to Br to I, we observe a corresponding decrease in both the low-temperature *χ*_M_*T* values and maximum magnetization values. This trend can be attributed to the influence of the halide ligand on the electronic structure of the Co(II) center through two primary mechanisms. Firstly, the more electronegative chloride ligand in **1Cl** withdraws electron density more strongly from the Co(II) center compared to bromide in **2Br** or iodide in **3I**. This creates a stronger ligand field effect that modifies the d-orbital splitting pattern and, therefore, leads to higher magnetic anisotropy (*D* = 25.3 cm^−1^) compared to **2Br** (*D* = 21.6 cm^−1^) and **3I** (*D* = 19.4 cm^−1^) [44]. Secondly, the covalency of the Co-X bond increases as we move from Cl to I, resulting in greater delocalization of electron density in the case of the Co-I bond compared to the Co-Cl bond. This increased covalency affects the spin–orbit coupling contribution to the magnetic anisotropy, as the more diffuse nature of the iodide orbital leads to reduced orbital contributions to the magnetic moment [15]. Consequently, complex **3I** exhibits lower *χ*_M_*T* values at low temperatures and reduced maximum magnetization compared to **1Cl**.

To further validate the magnetic anisotropy parameters obtained from experimental data fitting, we performed ab initio CASSCF calculations with ANO-RCC basis set approximations based on the crystal structures using the OPENMOLCAS package [45]. The calculated *D* values for complexes **1Cl**, **2Br**, and **3I** were found to be 30.5, 22.8, and 21.2 cm^−1^, respectively. These theoretical results are in good agreement with the values obtained from the PHI fitting of the experimental magnetic data, confirming the positive sign of the ZFS parameter *D* in all three complexes. Importantly, the computational results reproduce the same decreasing trend in *D* values across the series from chloride to iodide, providing strong theoretical support for our interpretation of the halide ligand effect on magnetic anisotropy.

### 3.3. AC Magnetic Measurements

The dynamic magnetic properties of complexes **1Cl**–**3I** were investigated using AC magnetic susceptibility measurements. In the absence of an external DC field, none of the complexes exhibited out-of-phase (*χ*″_M_) signals, indicating fast QTM and direct relaxation process at zero field (Figure S5) [12]. This behavior is common in Co(II)-based single-molecule magnets, where efficient fast relaxation processes often mask the slow relaxation of magnetization in zero field. The suppression of the QTM process is a critical consideration in the development of effective SMMs [46]. In SMM systems, relaxation pathways can be conceptualized as parallel circuits where the overall relaxation rate is dominated by the fastest relaxation process. QTM represents one of these fast relaxation pathways that can significantly undermine the desired slow magnetic relaxation behavior in zero field [12]. In our series of Co(II) complexes, while the half-integer spin nature of Co(II) should theoretically provide some protection against QTM, the presence of hyperfine interactions and other perturbations still allows for efficient tunneling in zero field.

**Figure 3:**
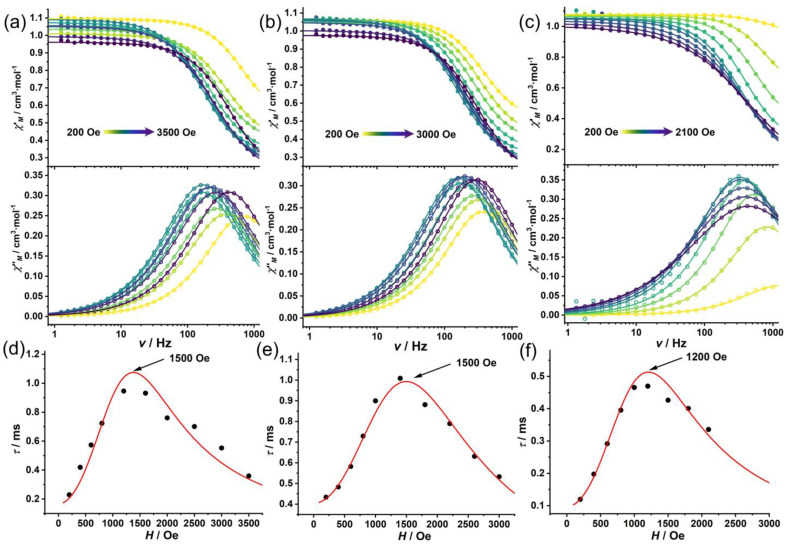
Field-dependent in-phase (*χ*′_M_, solid circles) and out-of-phase (*χ*″_M_, open circles) AC magnetic susceptibility plots, and field-dependent relaxation times for **1Cl** (**a**,**d**), **2Br** (**b**,**e**), and **3I** (**c**,**f**) under external DC magnetic fields. In a, b, and c, the circles and lines represent the experimental data and the fitting results using the generalized Debye model, respectively. In d, e, and f, the red solid lines represent the fitting results using Equation (1).

However, upon application of external DC fields, all three complexes displayed distinct temperature-dependent *χ*″_M_ signals, demonstrating that the static magnetic fields successfully suppressed the QTM process (Figure 3). The frequency-dependent AC susceptibility data were simultaneously fitted with the generalized Debye model (Equations (S1) and (S2)) [47] to extract the spin–lattice relaxation times (*τ*) at different temperatures (Figure 3d–f).

The field-dependent spin–lattice relaxation times exhibit a characteristic behavior: an initial increase in relaxation time at lower fields, indicating the progressive suppression of QTM, followed by a decrease at higher fields due to the growing contribution of the direct relaxation process [46]. At low fields, QTM dominates the relaxation process, providing a fast relaxation pathway through quantum mixing of states with opposite spin projections. As the field increases, this mixing is reduced because the Zeeman effect shifts the energy levels and removes the degeneracy at crossing points, effectively suppressing QTM. This explains the initial increase in relaxation time with the field. However, as the field continues to increase, the direct relaxation process (which scales with *H*⁴) becomes increasingly efficient. This process involves the direct emission of a phonon with energy equal to the Zeeman splitting between states, and becomes the dominant relaxation pathway at high fields, causing the relaxation time to decrease again. The field-dependent relaxation data were successfully fitted using Equation (1) (Table 4), where the first term describes the direct process, while the second term represents the QTM process and its field dependence [48]. From these analyses, optimal DC fields (*H_τ_*_,max_) of 1500, 1500, and 1200 Oe were determined for **1Cl**, **2Br**, and **3I**, respectively. In these fields, QTM is sufficiently suppressed while the direct process is not yet dominant enough to significantly accelerate relaxation [4].

(1) $$\tau^{-1}=AH^{2}T+\frac{B_{1}}{1+B_{2}H^{2}}$$

Based on the optimized DC fields, we conducted comprehensive temperature- and frequency-dependent AC magnetic susceptibility measurements for all three complexes. The Cole–Cole plots were fitted using the generalized Debye model, yielding *α* values below 0.22 for all complexes, indicating a narrow distribution of relaxation times.

All three complexes exhibit similar slow magnetic relaxation behavior, with the plots of ln*τ* vs. *T*^−1^ showing linear relationships in the high-temperature (low *T*^−1^) range. This initially allowed the data to be fitted using the Arrhenius law (Orbach process, Equation (2)), yielding *U*_eff_ = 21(1) K with *τ*_0_ = 1.1(4) × 10^−7^ s for **1Cl**, *U*_eff_ = 16.9(3) K with *τ*_0_ = 4.4(8) × 10^−7^ s for **2Br**, and 10.8(3) K with *τ*_0_ = 2.3(3) × 10^−6^ s for **3I**. However, the positive *D* values determined from our DC magnetic analysis indicate that the *M*_S_ = ±1/2 states lie lowest in energy, making thermal relaxation through excited *M*_S_ = ±3/2 states energetically unfavorable [49,50]. So, these complexes are unlikely to relax via a true Orbach process.

Ab initio calculations provide further insights into the relaxation dynamics of our Co(II) complexes. The calculated first excited Kramers doublets (KDs) for complexes **1Cl**, **2Br**, and **3I** lie at 78.2, 75.8, and 75.6 cm^−1^, respectively (Tables S4–S6). These values are significantly higher than the *U*_eff_ obtained from fitting the ln*τ* vs. *T*^−1^ data using an Orbach process. This discrepancy strongly suggests that faster relaxation processes dominate the magnetic relaxation in these systems. These likely include Raman processes, QTM, and direct relaxation processes. This conclusion is consistent with our field-dependent relaxation studies, which demonstrated the significant contribution of QTM in zero field and the increasing importance of direct processes at higher fields. Furthermore, examination of the relaxation data on a log–log scale (plots of *τ* vs. *T* in Figure S6) reveals non-linear behavior, indicating that the relaxation mechanisms cannot be attributed to a pure Raman process either.

**Figure 4:**
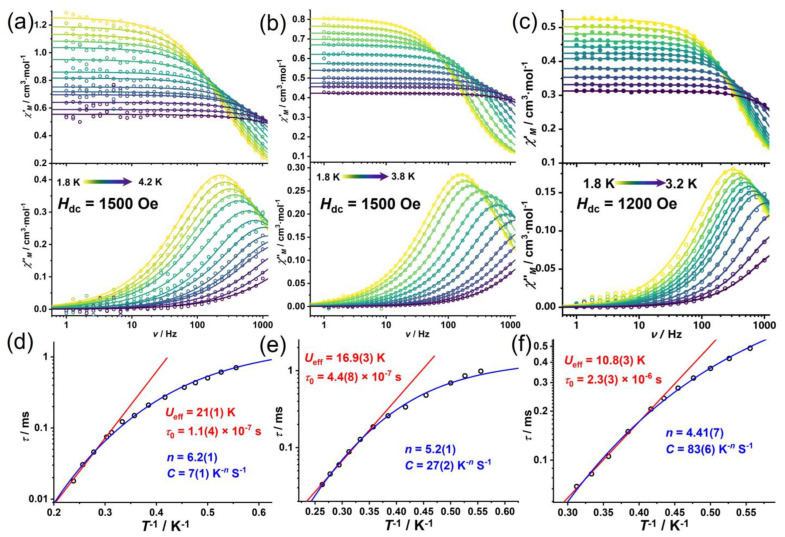
Frequency-dependent in-phase (*χ*′_M_, up) and out-of-phase (*χ*″_M_, down) AC magnetic susceptibility plots for complex **1Cl** (**a**), **2Br** (**b**), and **3I** (**c**) under the optimized DC fields. The circles and lines represent the experimental data and the fitting results using the generalized Debye model, respectively. Temperature-dependent relaxation time (open circles) and the fitting results (solid lines, red for the fit using Equation (2) and blue for the fit using Equation (3)) of complex **1Cl** (**d**), **2Br** (**e**), and **3I** (**f**).

Many studies have demonstrated that Co(II)-based SIMs often exhibit complex relaxation mechanisms dominated by direct, Raman, and QTM pathways rather than thermally activated Orbach processes [5,51,52]. For instance, the Co(II) complex [Co(acac)_2_(H_2_O)_2_] showed an apparent energy barrier of only ~16 cm^−1^ despite having a *D* value that would suggest a barrier of ~130 cm^−1^ if a true Orbach process were operating [49]. Therefore, we reanalyzed the temperature-dependent relaxation data using a more comprehensive model that combines direct, QTM, and Raman processes (Equation (3)). In this approach, the parameters for direct and QTM processes were fixed to the values obtained from the field-dependent analysis discussed earlier. This comprehensive analysis yielded satisfactory fits with Raman parameters of *n* = 6.2(1) and *C* = 7(1) K^−*n*^s^−1^ for **1Cl**, *n* = 5.2(1) and *C* = 27(2) K^−*n*^s^−1^ for **2Br,** and *n* = 4.41(7) and *C* = 83(6) K^−*n*^s^−1^ for **3I** (Figure 4f).

(2) $$\tau^{-1}={\tau_{0}}^{-1}\exp\left(-\frac{U_{eff}}{kT}\right)$$

(3) $$\tau^{-1}=AH^{2}T+\frac{B_{1}}{1+B_{2}H^{2}}+CT^{n}\;$$

Notably, there is a systematic decrease in the Raman exponent (*n*) from **1Cl** to **3I**, accompanied by an increase in the Raman coefficient (*C*). The variation in relaxation dynamics across the series can be attributed to the electronic influence of the different halide ligands. The more electronegative chloride in **1Cl** likely creates a stronger ligand field, resulting in larger energy separations between states and consequently slower relaxation compared to the less electronegative iodide in **3I** [53]. This suggests that the electronic effect of the chloride ligand dominates over the subtle structural variations in determining the relaxation dynamics [54]. The progressive shortening of relaxation times from **1Cl** to **3I** also correlates with the decreasing *D* values across the series, further supporting the critical role of magnetic anisotropy in determining the relaxation behavior of these complexes.

Many Co(II) complexes with positive *D* values (easy-plane anisotropy) exhibit slow magnetic relaxation behavior, which initially appears counterintuitive since traditional single-molecule magnet theory emphasizes the importance of negative *D* values (easy-axis anisotropy) for creating an energy barrier to spin reversal [49,55]. In such systems, the magnitude of *D* influences the matrix elements for QTM and direct relaxation processes that with larger *D* values, the ground state wavefunctions exhibit reduced mixing [56]. As well, the efficiency of both direct and Raman processes depends on the coupling between the spin system and the phonon bath, which is modulated by the spin–orbit coupling strength and consequently by the magnitude of *D*. For instance, the pseudotetrahedral cobalt(II) complex [(3G)CoCl]^+^ (3G = 1,1,1-tris- [2N-(1,1,3,3-tetramethylguanidino)methyl]ethane), with a positive axial zero-field splitting parameter of *D* = 12.7 cm^−1^, is shown to exhibit slow magnetic relaxation under an applied DC field [14].

Researchers suggest that relaxation from the *M*_S_ = +1/2 to the *M*_S_ = −1/2 level is slowed by a phonon bottleneck effect. Another explanation involves the role of transverse anisotropy in the xy plane. Under these conditions, the magnetization vector can be trapped in an “easy plane”, requiring energy to escape this configuration [52]. This mechanism has been observed in various Co(II) complexes, where the application of a small external magnetic field is often necessary to suppress quantum tunneling effects and reveal the slow relaxation behavior [57]. The ability to achieve slow magnetic relaxation in Co(II) complexes with positive *D* values significantly expands the structural and electronic landscape available for developing new single-molecule magnets with tailored properties.

## 4. Conclusions

In this study, we have synthesized and characterized three new pentacoordinate Co(II) complexes[CoX(dppb)_2_]X (X = Cl, Br, and I) with distinct halide ligands. Comprehensive structural and magnetic analyses reveal the systematic influence of the halide ligands on the magnetic properties of these complexes.

The structural characterization demonstrates that all three complexes adopt similar pentacoordinate geometries with vacant octahedron (*C*_4v_) coordination environments around the Co(II) centers. Magnetic studies confirm that all three complexes exhibit field-induced slow magnetic relaxation behaviors. Dynamic magnetic measurements show that the relaxation dynamics in these complexes involve a complex interplay of multiple mechanisms rather than a simple Orbach process. Comprehensive analysis of the relaxation data reveals that Raman processes dominate the relaxation at higher temperatures, with systematic variations in the Raman parameters across the series. The findings demonstrate that subtle electronic modifications through halide substitution can effectively influence the magnetic properties without substantially altering the coordination geometry. Future work could explore further electronic tuning through more diverse ligand substitutions, as well as detailed spectroscopic investigations to better understand the electronic structure of these complexes and their relationship to the magnetic properties.

## Figures and Tables

**Table 1:** Continuous Shape Measures Calculations for the Co(II) ions of **1**–**3**.

Ideal Structures			CShM Values for 1	CShM Values for 2	CShM Values for 3
PP-5	*D*_5*h*_	Pentagon	30.86784	31.19044	31.64043
vOC-5	*C*_4*v*_	Vacant octahedron	0.63020	0.80706	1.16116
TBPY-5	*D*_3*h*_	Trigonal bipyramid	5.76378	5.53698	5.15876
SPY-5	*C*_4*v*_	Spherical square pyramid	1.36270	1.19198	1.12414
JTBPY-5	*D*_3*h*_	Johnson trigonal bipyramid J12	8.01322	8.04524	8.03973

**Table 2:** The key differences in the coordination environments of complexes **1**–**3** and complex **4**.

Complex	P1-Co-P1 * Angle (°)	Dihedral Angle Between the Two Chelating P-Co-P Planes (°)	Distance Between the Co Ion and the P4 Basal Plane (Å)
**1Cl**	170.99(5)	9.05(5)	0.1108(7)
**2Br**	171.18(5)	8.85(5)	0.1071(8)
**3I**	170.54(7)	9.46(7)	0.1063(11)
**4**	169.02(3)	11.00(5)	−0.1296(8) **

* Symmetric code for **1**–**3**: 1/2 − *X*, +*Y*, 1/2 − *Z*; for **4**: 1/2 − *X*, 1/2 − *Y*, +*Z*. ** The negative value means the Co ion is under the P4 basal plane.

**Table 3:** Summary of fitting results of the DC magnetic data for complexes **1**–**3** using PHI and CASSCF computed values.

Complex	*D* (cm^−1^) ^[a]^	|*E*| (cm^−1^) ^[a]^	*g* ^[a]^	*D* (cm^−1^) ^[b]^	|*E*| (cm^−1^) ^[b]^
**1Cl**	25.3(2)	0.26(3)	2.42(2)	30.5	1.02
**2Br**	21.6(1)	0.22(2)	2.37(2)	22.8	0.64
**3I**	19.4(2)	0.21(3)	2.38(4)	21.2	0.58

^[a]^ SH parameters extracted from PHI fit. ^[b]^ Parameters obtained from CASSCF calculations.

**Table 4:** Summary of fitting results of the field-dependent AC magnetic data using Equation (3) for complexes **1**–**3**.

	*H_τ_*_,max_ (Oe)	*A* (s^−1^K^−1^ T^−2^)	*B*_1_ (s^−1^)	*B*_2_ (T^−2^)
**1Cl**	1500	1.15(7) × 10^4^	5.3(4) × 10^3^	596(28)
**2Br**	1500	1.09(3) × 10^4^	2.47(6) × 10^3^	149(11)
**3I**	1200	3.5(1) × 10^4^	1.03(3) × 10^4^	619(30)

## Data Availability

Data are contained within the article and Supplementary Materials.

## Supplementary Materials

The following supporting information can be downloaded at: https://www.mdpi.com/article/10.3390/molecules30112295/s1, Table S1: Crystal data and structure refinement for **1**–**3**; Table S2: Selected bond lengths (Å) and angles (°) for **1**–**3**; Table S3: *π*–*π* stacking parameters in complex **1**–**3**; Figure S1: Comparison of the coordination environments of [CoX(dppb)_2_]^+^ of the three compounds. The Co(II) ion and Co-X bonds coincide; Figure S2: Structure of the [CoCl(dppb)_2_]^+^ coordination cation of **1Cl** showing the intramolecular *π*–*π* stacking interactions. Hydrogen atoms are omitted for clarity. Color code: Co, turquoise; C, gray; P, pink; Cl, green; Figure S3: TG analyses for **1Cl** (top), **2Br** (middle), and **3I** (bottom); Figure S4: Experimental (black) and simulated (red) powder X-ray diffraction patterns of complexes **1** (top), **2** (middle), and **3** (bottom); Figure S5: Temperature-dependent in-phase (*χ*′_M_, solid circles) and out-of-phase (*χ*″_M_, open circles) AC magnetic susceptibility plots for **1Cl** (a), **2Br** (b), and **3I** (c) under a zero external magnetic field. The solid lines are only a guide for the eyes; Figure S6: The plots of *τ* vs. *T* on a log–log scale of the complexes **1** (top), **2** (middle), and **3** (bottom). The solid line is only a guide for the eyes; Figure S7. The IR spectra for the three compounds; Table S4. CASSCF computed spin-free and spin–orbit state energies for complex **1Cl**; Table S5. CASSCF computed spin-free and spin–orbit state energies for complex **2Br**; Table S6. CASSCF computed spin-free and spin–orbit state energies for complex **3I**.
