# Supplementary material for: Field-Induced Slow Magnetic Relaxation in Pentacoordinate Co(II) Complexes: Tuning Magnetic Anisotropy Through Halide Substitution
Source: Molecules. 2025 May 23;30(11):2295. doi: 10.3390/molecules30112295 (PMC12156324; doi:10.3390/molecules30112295)
Supplement: Supplementary file 1 [file molecules-30-02295-s001.zip › molecules-3659946-supplementary.pdf]

Supplementary Materials

Table S1. Crystal data and structure refinement for **1**-**3**.

	**1**	**2**	**3**
Empirical formula	$C_{62}H_{60}Cl_2CoO_4P_4$	$C_{62}H_{60}Br_2CoO_4P_4$	$C_{62}H_{60}CoI_2O_4P_4$
Formula weight	1122.81	1211.73	1305.71
Temperature/K	100.01(10)	99.99(10)	100.00(10)
Crystal system	monoclinic	monoclinic	monoclinic
Space group	*P*2/n	P2/n	P2/n
a [Å]	15.5129(9)	15.6992(4)	15.9678(10)
b [Å]	9.4572(5)	9.4890(2)	9.5904(6)
c [Å]	18.1791(11)	18.3492(4)	18.4554(11)
α [°]	90	90	90
β [°]	97.270(6)	98.138(2)	98.924(6)
γ [°]	90	90	90
Volume [Å^3]	2645.6(3)	2705.95(11)	2792.0(3)
Z	2	2	2
Density (calcd) [g cm^{-3}]	1.409	1.487	1.553
μ [mm^{-1}]	0.596	5.717	1.575
F(000)	1170.0	1242.0	1314.0
Radiation	MoKα (λ = 0.71073)	CuKα (λ= 1.54184)	MoKα (λ = 0.71073)
2θ range for data collection [°]	4.306 to 58.72	6.942 to 145.992	4.248 to 51.994
Reflections collected	13219	21484	12224
Independent reflections	6161 [R_{int} = 0.0628, R_{sigma} = 0.1058]	5292 [R_{int} = 0.0655, R_{sigma} = 0.0590]	5314 [R_{int} = 0.0510, R_{sigma} = 0.0710]
Parameters	311	304	304
Goodness-of-fit on F^2	0.992	1.071	1.035
Final *R* indexes [$I \geq 2\sigma(I)$]	R_1 = 0.0570, wR_2 = 0.1067	R_1 = 0.0408, wR_2 = 0.0997	R_1 = 0.0484, wR_2 = 0.1039
Final *R* indexes [all data]	R_1 = 0.0883, wR_2 = 0.1256	R_1 = 0.0530, wR_2 = 0.1063	R_1 = 0.0631, wR_2 = 0.1136

Table S2. Selected bond lengths (Å) and angles (°) for **1**-**3**.

Atom	Atom	Length/Å	Atom	Atom	Atom	Angle/°
**1**						
Cl1	Co1	2.3937(11)	P1	Co1	Cl1	94.51(2)
Co1	P1	2.2930(8)	$P1^1$	Co1	Cl1	94.51(2)
Co1	$P1^1$	2.2931(8)	P1	Co1	$P1^1$	170.99(5)
Co1	P2	2.2517(8)	P2	Co1	Cl1	91.05(2)
Co1	$P2^1$	2.2516(8)	$P2^1$	Co1	Cl1	91.05(2)
P1	C1	1.826(3)	$P2^1$	Co1	$P1^1$	81.98(3)
P1	C7	1.824(3)	P2	Co1	P1	81.98(3)
P1	C13	1.820(3)	$P2^1$	Co1	P1	97.85(3)
P2	C14	1.826(3)	P2	Co1	$P1^1$	97.85(3)
P2	C19	1.829(3)	$P2^1$	Co1	P2	177.89(4)
P2	C25	1.813(3)				
**2**						
Br1	Co1	2.5295(7)	$P1^1$	Co1	Br1	94.41(2)
Co1	P1	2.2875(6)	P1	Co1	Br1	94.41(2)
Co1	$P1^1$	2.2874(6)	$P1^1$	Co1	P1	171.18(5)
Co1	P2	2.2572(7)	$P2^1$	Co1	Br1	90.98(2)
Co1	$P2^1$	2.2572(7)	P2	Co1	Br1	90.98(2)

P1	C1	1.829(3)	P2	Co1	P1^{1}	97.83(3)
P1	C7	1.830(3)	P2^{1}	Co1	P1	97.83(3)
P1	C13	1.822(3)	P2	Co1	P1	82.02(3)
P2	C14	1.828(3)	P2^{1}	Co1	P1^{1}	82.02(3)
P2	C19	1.832(3)	P2^{1}	Co1	P2	178.05(5)
P2	C25	1.819(3)				
**3**						
I1	Co1	2.7014(9)	P1	Co1	I1	94.73(3)
Co1	P1 ^{1}	2.2923(12)	P1^{1}	Co1	I1	94.73(3)
Co1	P1	2.2922(12)	P1	Co1	P1^{1}	170.54(7)
Co1	P2	2.2692(12)	P2	Co1	I1	90.59(3)
Co1	P2^{1}	2.2691(12)	P2^{1}	Co1	I1	90.59(3)
P1	C1	1.826(5)	P2	Co1	P1	82.15(4)
P1	C7	1.825(4)	P2^{1}	Co1	P1	97.75(4)
P1	C13	1.827(4)	P2	Co1	P1^{1}	97.75(4)
P2	C14	1.829(5)	P2^{1}	Co1	P1^{1}	82.15(4)
P2	C19	1.832(5)	P2^{1}	Co1	P2	178.81(7)
P2	C25	1.820(5)				

Symmetric code: ^{1} 1/2-*X*, +*Y*, 1/2-*Z*.

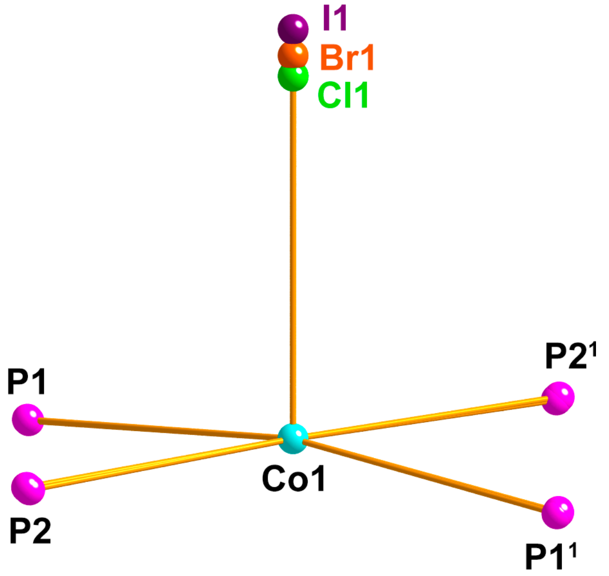

Figure S1. Comparison of the coordination environments of $[CoX(dppb)_2]^+$ of the three compounds. The Co(II) ion and Co-X bonds are coincide.

Table S3. π-π stacking parameters in complex **1**-**3**.

Members of the Rings	Complex	Plane-Plane Angle (°)	Centroid-to-Centroid Distance (Å)	Centroid-to-Centroid Shift (Å)
C11-C6, C19-C24	**1Cl**	4.942	3.838	2.059
	**2Br**	6.212	3.881	2.088
	**3I**	7.425	3.946	2.163
C7-C12, C25-C30	**1Cl**	10.715	3.521	1.504
	**2Br**	10.729	3.540	1.519
	**3I**	8.753	3.577	1.549
	**4**	10.393	3.499	1.244

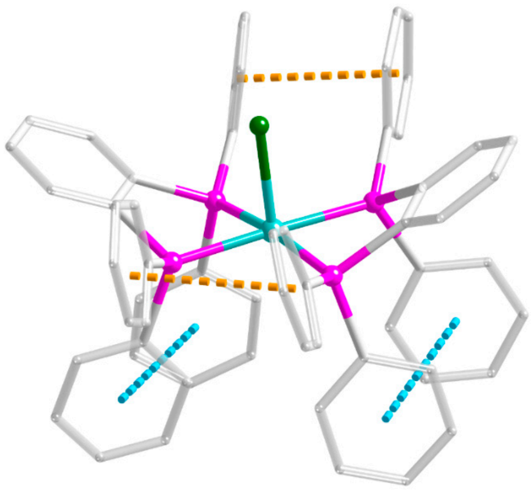

Figure S2. Structure of the $[CoCl(dppb)_2]^+$ coordination cation of **1Cl** showing the intramolecular π-π stacking interactions. Hydrogen atoms are omitted for clarity. Color code: Co, turquoise; C, gray; P, pink; Cl, green.

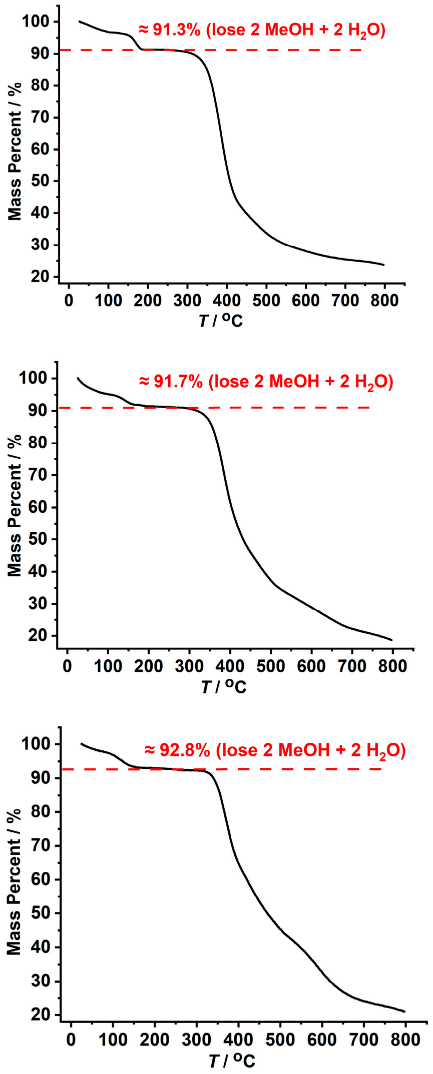

Figure S3. TG analyses for **1Cl** (top), **2Br** (middle) and **3I** (bottom).

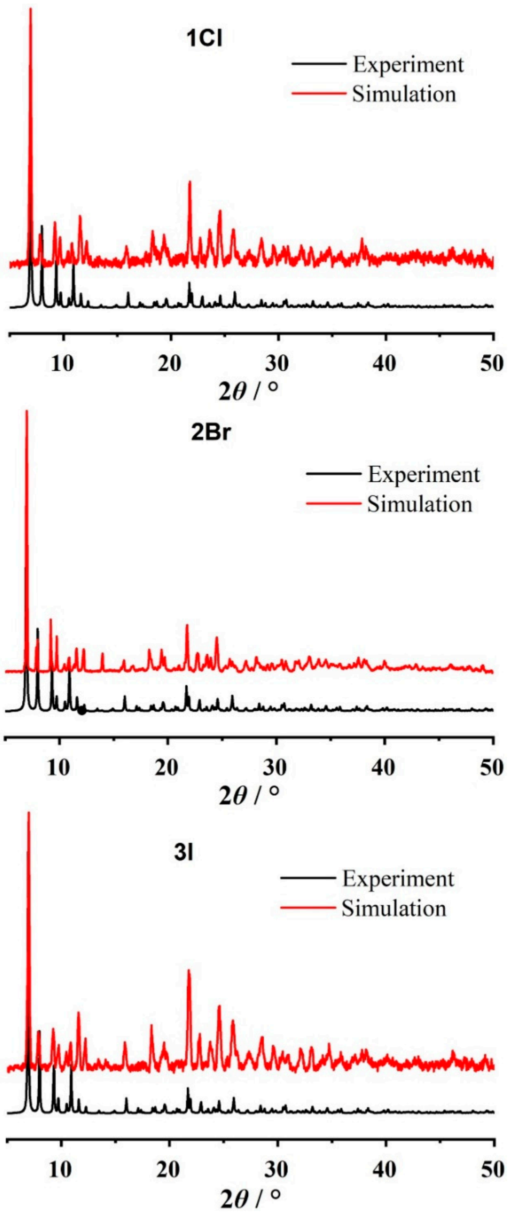

Figure S4. Experimental (black) and simulated (red) powder X-ray diffraction patterns of complexes **1** (top), **2** (middle) and **3** (bottom).

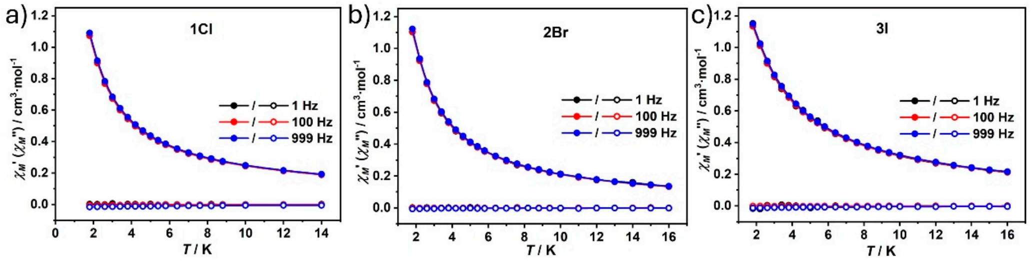

Figure S5. Temperature dependent in-phase (χ'_M, solid circles) and out-of-phase (χ''_M, open circles) ac magnetic susceptibility plots for **1Cl** (a) **2Br** (b) and **3I** (c) under a zero external magnetic field. The solid lines are only a guide for the eyes.

$$\chi' = \chi_s + (\chi_T - \chi_s)\frac{1+(\omega\tau)^{1-\alpha}\sin(\pi\alpha/2)}{1+2(\omega\tau)^{1-\alpha}\sin(\pi\alpha/2)+(\omega\tau)^{2-2\alpha}} \quad (S1)$$

$$\chi'' = (\chi_T - \chi_s)\frac{(\omega\tau)^{1-\alpha}\cos(\pi\alpha/2)}{1+2(\omega\tau)^{1-\alpha}\sin(\pi\alpha/2)+(\omega\tau)^{2-2\alpha}} \quad (S2)$$

In the equations, $\chi_S = \chi_{\omega\to\infty}$ is the adiabatic susceptibility, $\chi_T = \chi_{\omega\to 0}$ is the isothermal susceptibility, $\omega = 2\pi f$ is the angular frequency, α describes the distribution of relaxation pathways.

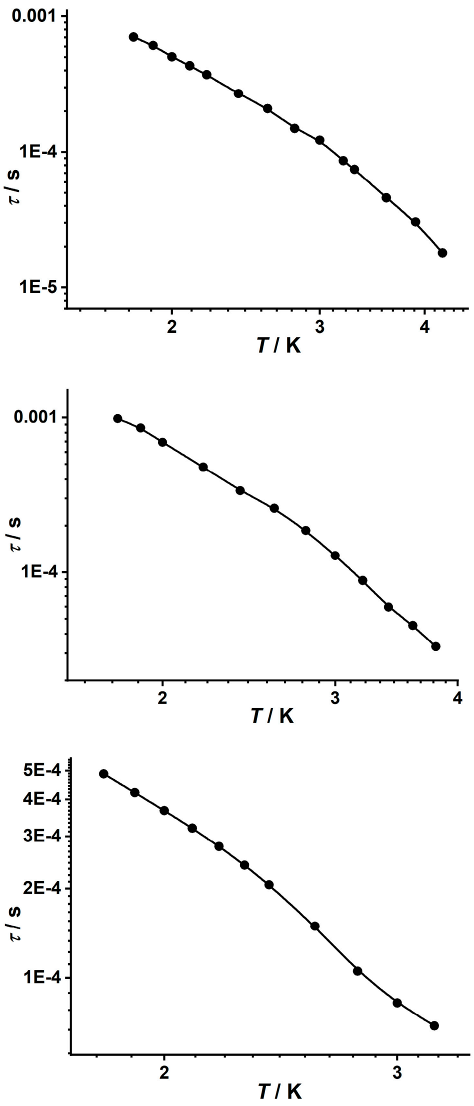

Figure S6. The plots of τ vs T on a log-log scale of the complexes **1** (top), **2** (middle) and **3** (bottom). The solid line is only a guide for the eyes.

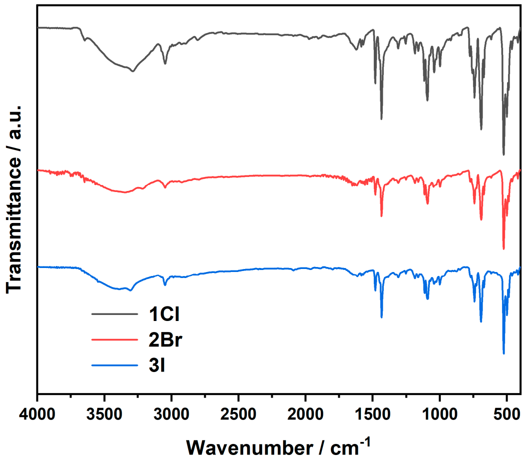

Figure S7. The IR spectra for the three compounds.

Ab Initio Calculations

All calculations were carried out with OPENMOLCAS version 20.10 and are of CASSCF/RASSI/SINGLE_ANISO type. The Cholesky decomposition threshold was set to 1×10^{-8} to save disk space. The Co(II) centers were calculated keeping the entire molecule and using the experimentally determined coordinates of atoms. We have employed the [ANO-RCC-VTZP] basis set for Co, Cl, Br, I, P, and the [ANO-RCC-VDZP] basis set for C and H.

Active space of the CASSCF method included 7 electrons in 5 orbitals (3d orbitals of Co(II) ion). All 10 quartets and 40 doublets were optimized in state-averaged calculations and then mixed by spin-orbit coupling. On the basis of the resulting spin-orbit states, SINGLE_ANISO program computed local magnetic properties (*g*-tensors, magnetic axes, local magnetic susceptibility, etc.)

Table S4. CASSCF computed spin-free and spin-orbit state energies for complex **1Cl**.

Spin-Free Energies (cm^{-1})	Spin-Orbit States (cm^{-1})
0	0
497.0453	0
1927.554	78.18326
2571.365	78.18326
10567.61	514.7099
11497.67	514.7099
19711.66	701.4867
24754.96	701.4867
24968.73	2167.693
30652.35	2167.693
	2242.525
	2242.525
	2816.391
	2816.391
	2921.848
	2921.848
	10744.05
	10744.05
	10771.9
	10771.9
	11684.84

	11684.84
	11729.39
	11729.39
	19929.88
	19929.88
	19932.81
	19932.81
	24920.03
	24920.03
	24945.89
	24945.89
	25164.64
	25164.64
	25205.99
	25205.99
	30854.6
	30854.6
	30865.09
	30865.09

Table S5. CASSCF computed spin-free and spin-orbit state energies for complex **2Br**.

Spin-Free Energies (cm^{-1})	Spin-Orbit States (cm^{-1})
0	0
485.2258	0
2130.339	75.80589
2727.877	75.80589
10927.35	508.4202
11808.97	508.4202
20170.1	693.3892
25074.2	693.3892
25195.89	2358.819
30555.62	2358.819
	2424.736
	2424.736
	2967.656
	2967.656
	3070.953
	3070.953
	11099.15
	11099.15
	11128.07
	11128.07
	11990.87
	11990.87
	12034.4
	12034.4
	20383.04
	20383.04
	20386.09
	20386.09
	25216.77
	25216.77
	25256.36

25256.36
25390.61
25390.61
25445.98
25445.98
30753.63
30753.63
30764.9
30764.9

Table S6. CASSCF computed spin-free and spin-orbit state energies for complex **3I**.

Spin-Free Energies (cm^{-1})	Spin-Orbit States (cm^{-1})
0.00000000000000	0.00000000000000
497.04588088322708	0.00000000000000
2285.64807257480152	75.61025013851372
2938.43874715575703	75.61025013851372
11087.99153892407048	521.89954953513961
12057.60993597990273	521.89954953513961
20304.43036334380668	705.92804226698127
25124.16647083594580	705.92804226698127
25266.43184352570097	2504.42223902428759
30191.58311617490835	2504.42223902428759
	2568.47641680776815
	2568.47641680776815
	3169.85516373652581
	3169.85516373652581
	3269.75529927836942
	3269.75529927836942
	11255.24083687912935
	11255.24083687912935
	11286.96156467138462
	11286.96156467138462
	12232.84116627957701
	12232.84116627957701
	12277.16908362954382
	12277.16908362954382
	20514.15601467198212
	20514.15601467198212
	20517.18694314155800
	20517.18694314155800
	25258.48777784408594
	25258.48777784408594
	25300.65036837458319
	25300.65036837458319
	25455.56741561765375
	25455.56741561765375
	25514.09433640387215
	25514.09433640387215
	30385.62099463670529
	30385.62099463670529
	30397.90115018612050
	30397.90115018612050
